# Valproic acid combined with cisplatin-based chemoradiation in locally advanced head and neck squamous cell carcinoma patients and associated biomarkers

**DOI:** 10.3332/ecancer.2020.1155

**Published:** 2020-12-15

**Authors:** Milena Perez Mak, Fatima Solange Pasini, Lixia Diao, Fabyane O Teixeira Garcia, Tiago Kenji Takahashi, Denyei Nakazato, Renata Eiras Martins, Cristiane Maria Almeida, Marco Aurelio Vamondes Kulcsar, Valdelania Aparecida Lamounier, Emily Montosa Nunes, Isabela Cristina de Souza, Marcio Ricardo Taveira Garcia, Alex Vieira Amadio, Sheila Aparecida C. Siqueira, Igor Moysés Longo Snitcovsky, Laura Sichero, Jing Wang, Gilberto de Castro

**Affiliations:** 1 Department of Medical Oncology, Instituto do Cancer do Estado de Sao Paulo, Hospital das Clinicas da Faculdade de Medicina da Universidade de Sao Paulo, Av Dr Arnaldo, 251 12th floor, CEP 01246-000, Sao Paulo, SP, Brazil; 2 Center for Translational Investigation in Oncology, Instituto do Cancer do Estado de Sao Paulo, Hospital das Clinicas da Faculdade de Medicina da Universidade de Sao Paulo, Av Dr Arnaldo, 251 12th floor, CEP 01246-000, Sao Paulo, SP, Brazil; 3 Department of Bioinformatics and Computational Biology, The University of Texas, MD Anderson Cancer Center, 1400 Pressler St. Floor 4, FCT4.6000, Houston, Texas, USA; 4 Head and Neck Surgery Department, Instituto do Cancer do Estado de Sao Paulo, Hospital das Clinicas da Faculdade de Medicina da Universidade de Sao Paulo, Av Dr Arnaldo, 251 12th floor, CEP 01246-000, Sao Paulo, SP, Brazil; 5 Department of Radiology, Instituto do Cancer do Estado de Sao Paulo, Hospital das Clinicas da Faculdade de Medicina da Universidade de Sao Paulo, Av Dr Arnaldo, 251 12th floor, CEP 01246-000, Sao Paulo, SP, Brazil; 6 Department of Pathology, Hospital das Clinicas da Faculdade de Medicina da Universidade de Sao Paulo, Av Dr Eneas de Carvalho Aguiar, 255, CEP 05403-000, Sao Paulo, SP, Brazil

**Keywords:** epigenetics, valproic acid, head and neck cancer, microRNA, chemoradiation

## Abstract

**Background:**

Cisplatin-based chemoradiation (CCRT) offers locally advanced head and neck squamous cell carcinoma (LAHNSCC) patients high local control rate, however, relapses are frequent. Our goal was to evaluate if association of valproic acid (VPA), a histone deacetylase (HDAC) inhibitor, with CCRT improved response rate (RR) and associated biomarkers.

**Methods:**

This phase II trial included patients with unresectable locally advanced (LA) oropharynx (OP) squamous cell carcinoma. CCRT began after 2 weeks of VPA (P1). Primary goal was RR at 8 weeks after chemoradiation (CRT)+VPA (P2). Biomarkers included microRNA (miR) polymerase chain reaction (PCR)-array profiling in plasma compared to healthy controls by two-sample t-test. Distribution of p-values was analysed by beta-uniform mixture. Findings were validated by real-time PCR quantitative polymerase chain reaction (qPCR) for selected miRs in plasma and saliva. p16, HDAC2 and RAD23 Homolog B, Nucleotide Excision Repair Protein (HR23B) tumour immunohistochemistry were evaluated.

**Results:**

Given significant toxicities, accrual was interrupted after inclusion of ten LA p16 negative OP patients. All were male, smokers/ex-smokers, aged 41–65 and with previous moderate/high alcohol intake. Nine evaluable patients yielded a RR of 88%. At false discovery rate of 5%, 169 miRs were differentially expressed between patients and controls, including lower expression of tumour suppressors (TSs) such as miR-31, -222, -let-7a/b/e and -145. miR-let-7a/e expression was validated by qPCR using saliva. A HDAC2 H-score above 170 was 90% accurate in predicting 6-month disease-free survival.

**Conclusions:**

VPA and CRT offered high RR; however, with prohibitive toxicities, which led to early trial termination. Patients and controls had a distinct pattern of miR expression, mainly with low levels of TS miRs targeting Tumor protein P53 (TP53). miR-let-7a/e levels were lower in patients compared to controls, which reinforces the aggressive nature of such tumours (NCT01695122).

## Background

The mainstay treatment for inoperable locally advanced head and neck squamous cell carcinoma (LAHNSCC) has been cisplatin-based chemoradiation (CCRT) for decades [1]. Although it provides adequate local control, relapses are frequent, especially in human papillomavirusIcesp: Instituto do Cancer do Estado de Sao Paulo (HPV)-negative oropharynx (OP) squamous cell carcinoma and with burdensome acute and late toxicities [2]. Over the years, newer strategies, such as neoadjuvant chemotherapy, altered fractionation radiation schemes and incorporation of targeted therapy drugs, such as cetuximab, have proven little to no improvement in long-term disease control or survival rates [3–5].

The association of HPV and OP squamous cell carcinoma is widely known to carry a better prognosis [6]. However, globally, disparities are seen regarding the incidence of HPV-associated LAHNSCC [7], and in developing countries, such as Brazil, most of these tumours are associated with high intake of alcohol and tobacco.

Epigenetic regulation plays a pivotal role in tumour progression and therapy response. Histone deacetylase inhibitors (HDACis), such as vorinostat, have already proven therapeutic efficacy in leukaemia and are currently in use for the treatment of cutaneous T-cell lymphoma [8]. Valproic acid (VPA) has long been used as an anti-epileptic drug and mood stabilizer and has a well-known toxicity profile [9]. It is widely available and with an accessible cost, which makes it attractive to study as therapeutic repurposing. VPA is a pan-HDACi, and encouraging pre-clinical data have demonstrated its anticancer potential [10–13]. Numerous small clinical trials have evaluated VPA in combination with other epigenetic regulators such as 2′-deoxy-5-azacytidine (AZA) or standard treatment as chemotherapy or radiation both in haematological and solid tumours, with conflicting results (Table 1) [14–22].

The therapeutic activity of HDACi has been associated with altered expression of HDAC2 and HR23B [23–25]. Moreover, other associated biomarkers, such as microRNAs (miRs) are also under study [26, 27]. Previous research has shown that miRs are also trafficked through exosomes, exerting oncogenic potential with increase in invasiveness and phenotypic changes in cancer cell lines [28]. Secretion of exosomes by cancerous tissue can be identified in numerous biofluids, such as saliva, which can be easily collected from patients.

This phase II trial was designed to evaluate the efficacy of the association of VPA to standard CCRT in patients with OP LAHNSCC and associated biomarkers.

## Methods

### Study design and patients

This single-arm phase II trial included locally advanced (LA) OP cancer patients treated at a single institution (ICESP). Eligible patients were under 65 years-old, had unresectable LA OP cancer and were candidates to definitive CCRT. Patients had an Eastern Cooperative Oncology Group (ECOG) performance status of 0–2 and adequate renal, haematological and liver functions as specified in the study protocol. Measurable radiological disease as defined by the Response Evaluation Criteria in Solid Tumours (RECIST) 1.1 was mandatory. Patients treated with anti-epileptic drugs, previous use of VPA, with hypoalbuminaemia, positivity for human immunodeficiency virus or active hepatitis B, C were excluded.

Our primary goal was to evaluate CCRT combination with VPA response rate (RR) at 8 weeks post-treatment as assessed by RECIST v1.1. Secondary goals were to evaluate treatment related toxicities per National Cancer Institute’s Common Terminology Criteria for Adverse Events v.4.0, disease-free survival (DFS), evaluated as time from the beginning of treatment until progression or death from any cause, overall survival (OS) and quality of life.

### Study treatment

Eligible patients were given VPA from 2 weeks before starting CCRT until the last fraction of Radiotherapy (RT). VPA was started at 15 mg/kg/day orally and adjusted to a therapeutic plasma level of 40–100 mcg/mL. CCRT consisted of cisplatin 100 mg/m^2^ d1, d22 and d43 and definitive 70 Gy 3D RT in 30 fractions. After the inclusion of the first ten patients, given the toxicity profile, the protocol was amended, and patients were given cisplatin on d1 and d22 and VPA was omitted on the first and third weeks of RT.

Biopsy tissue was analysed for p16, HPV, HDAC2 and HR23B. Plasma H3 and H4 histone acetylation in peripheral monocytes was evaluated at baseline (P0), 2 weeks after VPA (P1) and at the end of RT (P2). miR profiling was performed in plasma samples from P0, P1 and at the time of response evaluation, P3. Saliva was analysed at P0, P1 and P3 to validate relevant miRs found in plasma. Extracellular vesicles (EVs) were extracted from patients and healthy volunteers (HV) plasma. HV comprised by smokers and former smokers were submitted to miR plasma profiling and saliva analysis. EV from patients and HV were added to HNSCC cell lines to evaluate cisplatin sensitivity and cell migration.

### Statistical design

This trial was a two-step phase II Simon design. Given a RR of 60% in the historical control group [29] and 80% for the experimental group, the inclusion of 40 patients was foreseen, with alpha = 5% and beta = 20%. The study would continue to the second step if 8 of 13 responses were found in the first step and would be considered positive if 25 of 35 responses were found overall. DFS and OS were estimated by Kaplan–Meier (IBM Statistical Package for the Social Sciences (SPSS) Statistics for Windows, Version 20.0. Armonk, NY: IBM Corp.). Efficacy analyses were intention-to-treat based and safety analysed for all patients that received at least 1 week of concurrent VPA and radiotherapy. A safety interim analysis was planned after the first stage (13 patients).

This study was approved by the local ethics committee and registered in ClinicalTrials.gov (NCT number): NCT01695122. Informed written consent was obtained from all trial participants.

### Tissue p16, HR23B and HDAC2 immunohistochemistry and HPV testing

Patients’ archival tissue from initial diagnosis was analysed to determine p16, HR23B, HDAC2 immunohistochemistry expression. p16 status was determined by anti-p16 antibody clone 6175-405 (Zeta, 1:400). Positive expression required over 70% of cells with strong and diffuse nuclear and cytoplasmic expression. Nuclear positivity for anti-HR23B (clone Ab 88503; Abcam®) and anti-HDAC2 (clone Ab 16032; Abcam®, MA, USA) antibodies was quantified with an H-score. Correlation with disease control rate at 6 months was determined by receiver operating characteristic (ROC) curve performed by XLSTAT™ version 2016.05.34949 software. HPV testing was performed by a polymerase chain reaction (PCR) multiplex assay (Luminex, Luminex Corp., Austin, TX, USA).

### Peripheral monocytes H3 and H4 acetylation

Peripheral monocytes acetylation was analysed in patients’ plasma samples at baseline, 2 weeks after VPA and at the end of chemoradiation. Samples were not collected when VPA was discontinued. EpiQuik™ Global Histone H3 Acetylation Assay Kit (Epigentek Group Inc., NY, USA) was utilized. Samples were processed as per package instructions. Total protein expression was determined by Molecular Probes—Qubit® Protein Assay Kits (Invitrogen, CA, USA). The ratio between samples and blanks adjusted by the angular coefficient was used to determine the acetylation index. Assays were performed in triplicates.

### miR profiling

Unstimulated saliva was collected from patients and HV. Plasma samples were collected at the designated time points (P0, P1 and P3). Processing was done through successive cold centrifugation and total RNA was extracted with miRNeasy Mini Kit (Qiagen, Hilden, Germany) following package instructions. RNA concentration was determined through spectrophotometry with NanoDrop 1000 (Thermo Fisher Scientific, CA, USA). Plasma miR profiling was done by qPCR with TaqMan® Human microRNA Array v2.0 (Applied Biosystems, CA, USA). To analyse results, cycle threshold (Ct) values were determined with Real Time SDS® Software (Applied Biosystems, CA, USA). Statistical analysis was performed using R (https://www.r-project.org/), a publicly available statistical computing software. Data was normalised and analysed by the comparative Ct method [30]. A two-sample *t*-test was applied to each of the miRs. To correct for multiple hypotheses testing, the resulting *p*-values were modelled by a beta-uniform mixture (BUM) model. To identify differentially expressed miRs, cutoffs were determined by controlling the false discovery rate (FDR) [31–33].

Selected miRs were assayed in triplicate by RT-qPCR with miRNA TaqMan® Advanced (Life Technologies Corporation, CA, USA) assay. Ct values were determined with StepOne v2.3 (Applied Biosystems, CA, USA) software. Samples were normalised with Normfinder algorithm and the most stable miRs were selected to normalise samples. miR-26b-5p was the internal normaliser for plasma samples and miR-21 for saliva samples and final values were expressed as 2^−∆∆Ct^.

Top differentially expressed miRs were evaluated by pathway analysis with Diana miRPath_v3 [34]. Kyoto Encyclopedia of Genes and Genomes (KEGG) analysis was performed with validated miR targets (DIANA-TarBase miRPath v.3).

### EVs extraction and cell-line functional assays

EVs were isolated from cells and debris with serial centrifugation. Quantification and size distribution were determined with Malvern NanoSight LM14 (Malvern Panalytical, Malvern, UK) and EV denomination was done by sizing. Head and neck cell-lines SCC-4 (CRL-1624), SCC-9 (CRL-1629) and SCC-25 (CRL-1628) originated from the American Type Culture Collection. Cells were cultured with Dulbecco’s modified Eagle and HamF12 (1:1) medium. To determine cisplatin sensitivity (IC50) MTT assays were performed at 24 and 48 hours with increasing cisplatin concentrations (5 to 100 μM), in triplicates. Next, EVs were added at a ratio of 10^8^ particles/ml for each 10^4^ cells. Fetal bovine serum was depleted from EV. A pool of EV from each group (HV, responders at P0 and non-responders at P0) was added to each cell line, and the half maximal inhibitory concentration (IC50) doses of cisplatin were evaluated after 24 and 48 hours. Cell-line migration (SCC-9) was assessed by wound healing assay. Cells were cultured in an EV-free medium up to 90% confluence. EV from HV and responders and non-responders at different time points was added. Hourly photos were taken with Invitrogen^TM^ EVOS^TM^ FL Auto Imaging System (Thermo Fisher Scientific, CA, USA). Image J was used to quantify cell migration rate.

Statistical analysis comprised of parametric *T*-test and Analysis of variance (ANOVA) was performed with IBM SPSS Statistics for Windows, Version 20.0. Armonk, NY: IBM Corp.

## Results

**Figure 1.**
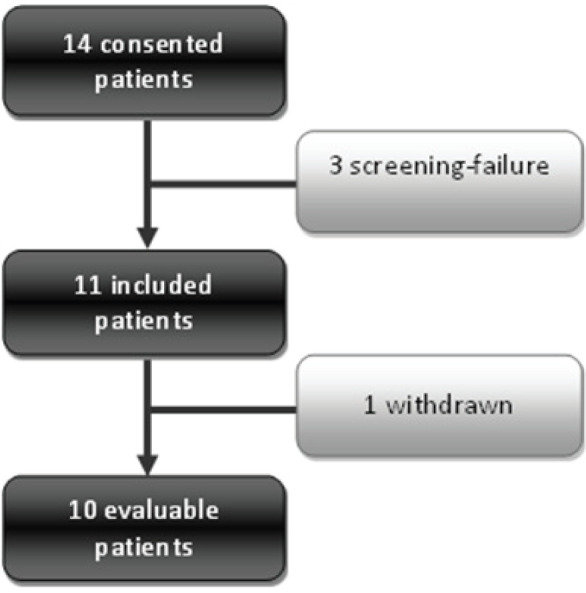
Consort diagram. Consort diagram of patients included in the study from 2012 to 2014. One patient was withdrawn due to performance status deterioration before CCRT.

Fourteen patients were included in this study from September 2012 to June 2014. There were three screening failures and 11 were included and treated. One patient was withdrawn given performance status deterioration before CCRT, and one patient was unevaluable at the time of response assessment (Figure 1).

Patient characteristics are summarised in Table 2. All patients were male, had LA OP cancer, were current or ex-smokers and former alcoholics. Median age was 55 years-old and half the patients had a weight loss above 10% in the 6 months before inclusion. Although all patients were p16 negative, high-risk HPV was detected in three patients (one HPV18, one HPV18 and HPV16 and one HPV56).

After the inclusion of the first ten patients, an interim safety analysis was performed. Three patients had been hospitalised, two in critical care units due to renal failure, respiratory infection and syncope. The protocol was emended and VPA was omitted on the week that cisplatin was administered and only two cycles of cisplatin were given concurrently with radiotherapy (d1 and d22). Two more patients were included and experienced grade 3 and 4 adverse events (disseminated herpes zoster and radiodermitis, respectively). Given the high rate of grade 3/4 adverse events (8 out of 9 patients), we interrupted the trial to preserve participants’ safety (Table 3).

**Supplementary Figure 1.**
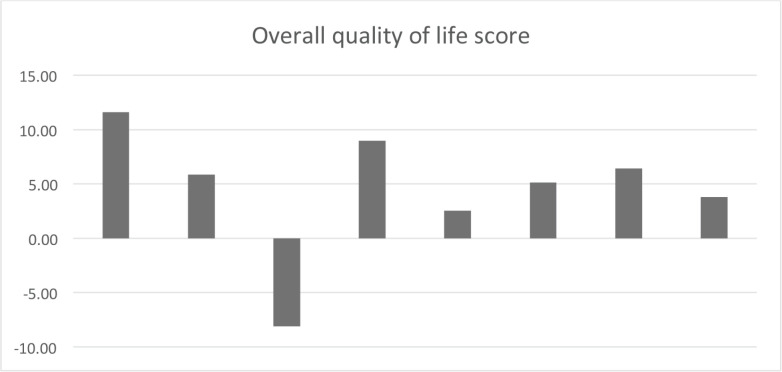
Baseline versus end-of-treatment variation of the overall quality of life score. The majority of patients had an improvement in quality of life at the moment of response evaluation (>5 points). EORTC QLC-30.

In the intention-to-treat population a median of 230 mg/m^2^ of cisplatin was administered and all patients received 70 Gy (Table 2). VPA mean plasma concentration was of 42 mcg/mL and 80% of the patients achieved the target concentration of 40–100 mcg/mL. The primary goal of the phase II first step analysis was reached. A total of eight patients had a complete response (CR) or partial response (PR) (five and three patients, respectively) as seen in Table 4. Most patients reported improvement in quality of life (Supplementary Figure 1). At a median follow-up of 52 months, six patients experienced treatment failure, with two systemic progressions. Currently, five patients are alive and without evidence of disease, with a median OS of 44 months (Table 4).

## Exploratory results

### HDAC2 immunohistochemistry (IHC) expression is related to prognosis

**Supplementary Figure 2.**
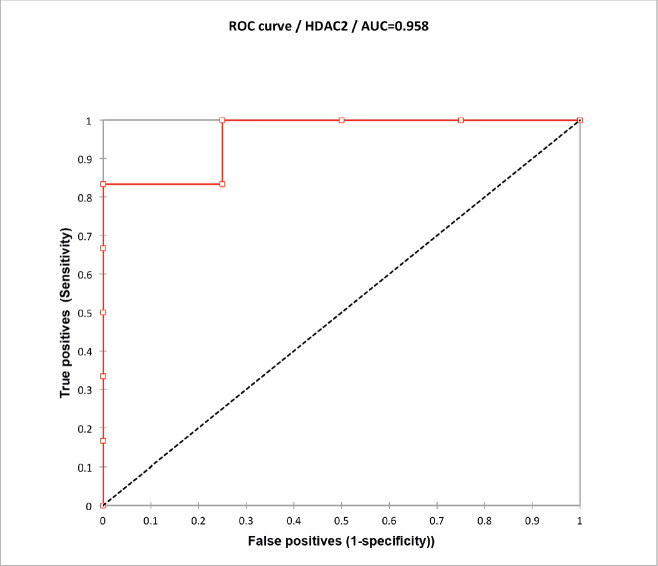
ROC curve analysis of HDAC2 H-score. Values over 170 correlated with DFS at 6 months with a sensitivity of 83% and specificity of 100%.

HDAC2 mean H-score was 164 (80–230) and HR23B 176.5 (95–240). A ROC curve analysis was performed correlating the expression levels with DFS at 6 months (DFS6). An HDAC2 H-score higher than 170 had a 90% accuracy at predicting DFS6 with an area under the curve of 0.958. HR23B H-score was non-informative (Supplementary Figure 2).

### miR profiling differs in patients and HV

In order to determine possible diagnostic and prognostic miRs in this group of patients, miR profiling analysis was performed. A cohort of seven smokers or ex-smokers HV was used as a control group. We considered responders as patients who were disease free at 6 months. Patients’ pre-treatment plasma profiling (P0) was compared to control, after 2 weeks of VPA (P1) and post-treatment (8 weeks after CCRT+VPA, P3). We also compared the profile of responders versus non-responders at baseline, responders at P0 versus P3 and non-responders at P0 versus P3.

**Supplementary Figure 3.**
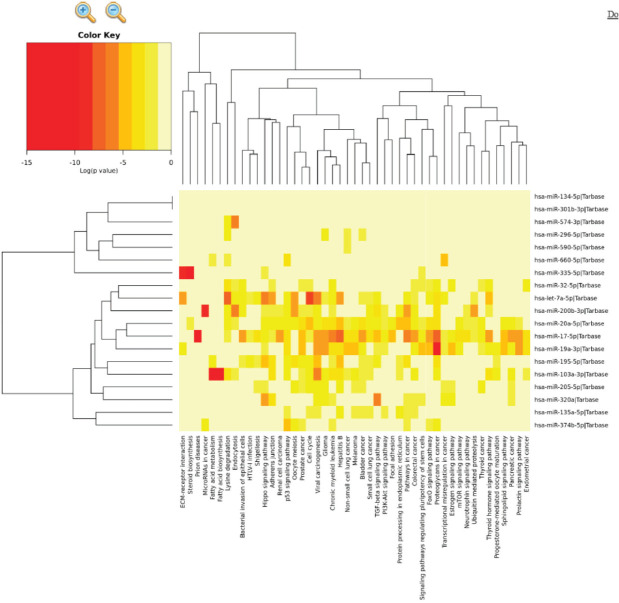
Pathway analysis of differentially expressed miRs between responders and non-responders at baseline. Diana miRPath version 3 KEGG pathways of 19 differentially expressed miR targets.

**Figure 2.**
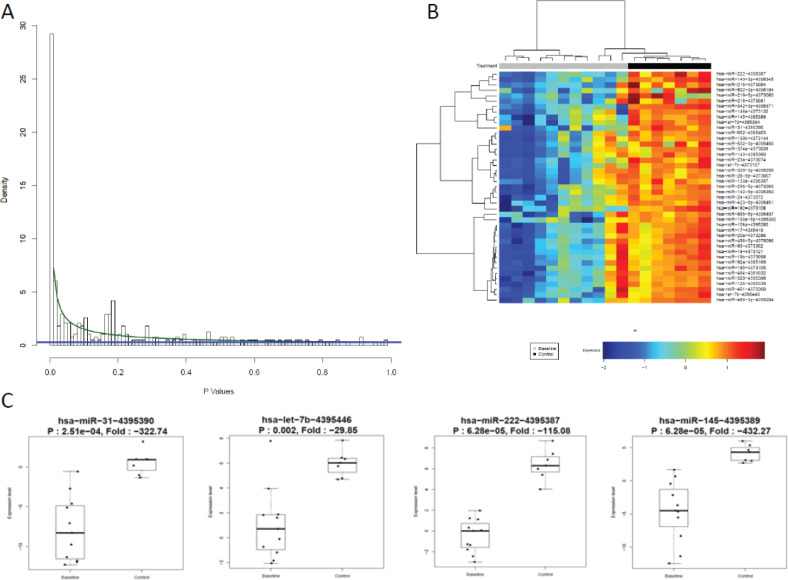
miR expression differs between controls and patients at baseline. (a): BUM analysis showed that a great number of miR were differentially expressed between patients and HV. At an FDR of 5%, 169 miRs were found to be relevant, *p* cutoff of 0.065. (b): Heat map of top differentially expressed miRs by two-way hierarchical clustering (miRs correlation by Pearson). Cancer patients underexpressed several miRs compared to HV. (c): Top miRs which were underexpressed in patients compared to volunteers are TS miRs.

**Figure 3.**
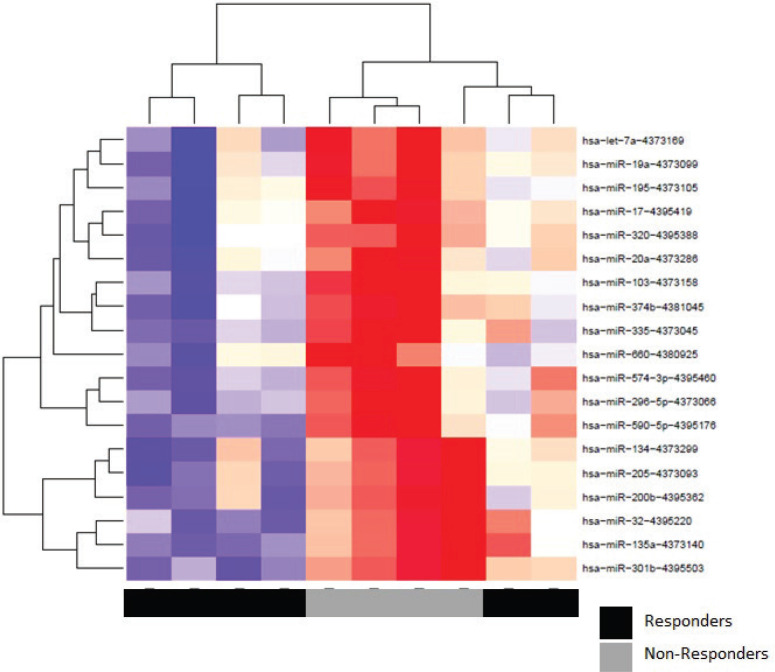
Heat map of hierarchical clustering analysis of miR expression (Pearson correlation) of responders and non-responders.

Patients at baseline compared to controls had several differentially expressed miRs. At an FDR of 5%, 169 miRs had a statistically significant difference in expression (*p* = 0.065). Notably, several tumour suppressor (TS) miRs were underexpressed, such as miR-31, -let-7b/e -145 (Figure 2, Table 5). At baseline, responders and non-responders also had different miR pattern expression. At an FDR of 5%, 19 miRs were found to be statistically significant, as seen in Figure 3. Top miR was miR-200b, which had a higher expression in responders (*p* = 0.002). Other downregulated miRs in non-responders included tumour suppressive miRs-103, -374b, -let-7a and epithelial-to-mesenchymal transition-related miR-205 [35–37]. Pathway analysis showed enrichment in pathways related to cell adhesion and migration, with ‘Proteoglycans in cancer’ and ‘Hippo signaling pathway’ being the top KEGG pathways identified (Supplementary Figure 3).

Patterns of miR expression profiles were not significantly different in the other analysis performed.

Next, we sought to validate the miR profiling findings with RT-PCR using plasma and saliva. Relevant miRs were selected through annotation. Plasma median expression of miRs throughout the different groups was compared. Undetermined readings were excluded. Unexpectedly, miR-1-3p and -let-7e, were found to have a higher median expression in patients than in HV (Supplementary Table 1). Seeking to evaluate a different methodology to assess miR expression, we determined median expression of miRs-1-3p, -let-7a-5p, -7e-5p, -32-5p, 660-5p in saliva. End-of-treatment samples were not evaluated, since intense xerostomia precluded most of the patients from providing adequate samples. In agreement with taqman low density array (TLDA) profiling, miR-let-7e and -let-7a also had a higher expression in controls to patients at baseline (Table 6).

### Patient derived EVs increase cell migration

**Figure 4.**
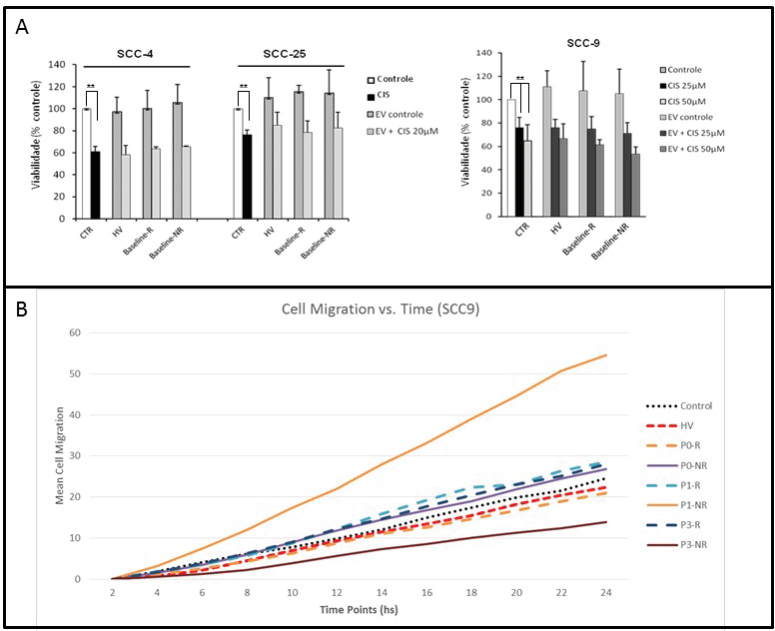
Functional assays using EVs. (a): Cisplatin IC50 was determined for three HNSCC cell lines (SCC-4, SCC-25 and SCC-9). Control samples had a statistically significant decrease in cell viability with cisplatin in 3-(4,5-Dimethylthiazol-2-yl)-2,5-diphenyltetrazolium bromidefor (MTT) assays. The addition of HV, responders and non-responders EVs did not alter significantly cisplatin cell sensitivity. Values are means ± SD of three separate experiments. (b): Scratch migration assay with SCC-9. Assays with EV from HV, responders at P0, P1 and P3 (hashed lines) and non-responders at P0, P1 and P3 (straight lines) were compared to controls. EV from non-responders led to a significant increase in SCC-9 migration rate compared to control (*p* < 0.001). Values are means ± SD of at least two separate experiments.

We sought to evaluate patient derived EVs impact on head and neck cancer cell lines sensitivity to cisplatin and migration. When analysing cell lines’ (SCC4, SCC9 and SCC25) sensitivity to cisplatin, no effect was observed with the addition of EV derived from HV, responders at baseline or non-responders at baseline (Figure 4a). Scratch migration assay with SCC-9 demonstrated an increased migration rate with the addition of EV from non-responders after 2 weeks of VPA compared to control (*p* < 0.001) (Figure 4b).

## Discussion

In this phase II trial of the role of epigenetic regulation in the standard treatment of cisplatin-based CRT in LAHNSCC, we were able to show an apparent increase in RR, since our goal of eight responses in the first stage of Simon’s design was met. Unfortunately, given the unacceptable toxicity encountered, we were not able to complete the trial. Patients submitted to CCRT and VPA experienced a higher rate of medullary toxicity and infection than reported in the literature and historical control data from our institution [29]. A possible synergistic effect of VPA and CCRT in the observed lymphopenia might have occurred, leading to the exacerbated toxicity in this trial [38, 39]. Somnolence was frequent, however, manageable and there was no apparent compromise in quality of life. Toxicity and efficacy results found in our study are similar to that reported in a phase I trial of HDACi vorinostat combination with CCRT in LAHNSCC patients. Although a high RR of 96.2% was found, 65% of patients experienced grade 3/4 lymphopenia [40].

One interesting finding is the detection of high-risk HPV DNA in three patients, even though none presented positivity for p16. Epidemiological research has shown a low-prevalence of HPV-related OP cancer in Brazil [7]. Moreover, the high exposure of other risk factors in this population (smoking and drinking habits) might explain the negativity of p16. This suggests an alternate carcinogenesis in these patients other than inactivation of p53 and pRb HPVs E6 and E7 oncoproteins, respectively, and explains a prognosis similar to the rest of the patients included in this trial. Furthermore, as HPV mRNA was not accessed, we are unable to ascribe these tumours as HPV-induced.

An HDAC2 H-score above 170 had an apparent correlation with DFS in this population, suggesting its prognostic role. Although overexpression of this marker has been previously correlated with poorer survival in other tumour types [23, 25], the use of VPA in this population might have had an influence in the observed data. However, given the small sample size, conclusions are limited.

We were unable to evaluate the *in vivo* effect of VPA through the assays performed to evaluate peripheral monocytes acetylation (PMAC). PMAC was assessed at baseline (P0), 2 weeks after VPA (P1) and at the end of CCRT (P2). Considering a fold-change cutoff of two, only one patient demonstrated a change in H3 and H4 acetylation through the assay employed (data not shown). However, patients had adequate serum levels of VPA and experienced increased local toxicities such as radiodermitis and mucositis, suggesting that a possible radiosensitising effect with the association of VPA to CCRT did occur. Previous studies have also failed to demonstrate differences in PMAC with VPA [41], given its major effect in HDAC2 [25]. Moreover, the methodology employed might also have an effect in the evaluation of PMAC, given different results obtained in other studies with the use of Western blotting and immunofluorescence [25, 42, 43]. Finally, other studies showed that PMAC has no apparent correlation with responses to VPA, given its broad mechanism in histone deacetylase inhibition [44, 45].

The miR profiling demonstrated a marked difference in the pattern of miR expression in patients and HV. LAHNSCC patients underexpressed various TS miRs at baseline compared to HV, suggesting that miR regulation might be lost during cancer progression. Some of the miRs that were identified (miRs-31, -let-7b/e, -222, -145) target known deregulated genes either by mutation or epigenetic regulation (such as promoter methylation) in alcohol and tobacco related HNSCC, such as *TP53, MET Proto-Oncogene, Receptor Tyrosine Kinase (MET), Cyclin D2EGFR: epidermal growth factor receptor (CCND2), EGFR, transforming growth factor beta (TGFB), Cyclin Dependent Kinase 6 (CDK6)* and Vascular Endothelial Growth Factor (*VEGFA)*. Moreover, 19 miRs were differentially expressed between responders and non-responders at baseline, suggesting a prognostic role of miRs in this population, which could be validated in future studies. Interestingly miR-let-7a was also underexpressed in non-responders compared to responders at baseline in the miR baseline plasma profiling. Additionally, there was no apparent miR regulation with the use of VPA or during treatment.

We sought to validate our plasma miR profiling findings for selected miRs with RT-PCR using plasma and saliva. Although we were unable to do so in plasma samples, a concordant finding regarding differential expression of miR-let-7a/e in patients compared to HV in saliva was found. miR-let-7 family has been found to be underexpressed in dedifferentiated tumours and associated with epithelial-to-mesenchymal transition [46, 47]. Moreover, given its TS role, it is also underexpressed in larynx cancer [48, 49]. Finally, miR-let-7e expression has also been associated with a worse prognosis in tongue and lung cancer patients [50, 51]. Collectively, these observations suggest the diagnostic role of miR-let-7a/e, which can be evaluated in saliva as demonstrated here.

Functional assays with HV and patient-derived EVs suggested an increased aggressiveness profile in non-responders EV, given the higher migration rate found with the addition of EV from non-responders to head and neck cancer cell line SCC-9.

## Conclusions

Our study suggests that incorporation of deacetylase inhibition in the standard treatment of LAHNSCC increases CCRT RR; however, given the toxicity observed here, the combination of VPA with CCRT is not recommended. We were able to characterise a distinct pattern of miR expression in patients and HV, as well as a differential expression in responders and non-responders, which further underscores miRs as diagnostic and prognostic biomarkers in LAHNSCC. Validation of miR-let-7a/e in saliva as a diagnostic marker poses an interesting assessment in the clinic, avoiding an uncomfortable puncture. Future studies addressing the role of histone acetylation inhibition are warranted to attain better results in the treatment of this LAHNSCC. Novel combinations with less toxic therapies, such as immunotherapy [52], may allow the incorporation of epigenetic regulation to a broader patient population.

## Declarations

### Ethics approval and consent to participate

The study was approved by the Institutional Review Board of the Faculdade de Medicina da Universidade de São Paulo under number 327/11. All participants provided written informed consent.

### Consent for publication

Study participants signed informed consent to publish the results in peer reviewed journal. The team also has consent to publish study findings.

### Availability of data and materials

The datasets generated and/or analysed during the current study are not publicly available due to patients’ confidentiality but are available from the corresponding author on reasonable request.

## Competing interests

The authors declare that they have no competing interests.

## Funding

This study was funded by FAPESP: 2015/01584-1; 2014/26965-5. The funding source had no involvement in study design; in the collection, analysis and interpretation of data; in the writing of the report; or in the decision to submit the article for publication.

## Authorship contributions

**Study concepts:** MPM, FSP, IMS, GCJ

**Study design:** MPM, FSP, IMS, GCJ

**Data acquisition:** MPM, FOTG, TKT, DN, REM, CMA, MAVK, EMN, ICS, MRTG, AVA

**Quality control of data and algorithms:** MPM, FSP, LD, SACS, LS

**Data analysis and interpretation:** MPM, FSP, LD, AVA, LS, JW

**Statistical analysis:** FSP, LD, JW

**Manuscript preparation:** MPM, FSP, LD, JW, GCJ

**Manuscript editing and review:** All authors have read and approved the manuscript.

## Figures and Tables

**Supplementary Table 1.** Mean expression of selected microRNAs for qPCR plasma validation for each study group.

	Mean control (*N*)	P0 median (*N*)	P1 median (*N*)	P3 median(*N*)	*p*
miR-1-3p	0.25 (7)	10.73 (11)	11.66 (11)	20.87 (10)	0.001
miR-let-7a-5p	1.62 (7)	4.09 (11)	4.54 (11)	3.52 (10)	0.13
miR-let-7e-5p	0.67 (7)	11.66 (11)	9.47 (11)	13.40 (10)	0.003
miR-17-5p	2.30 (7)	1.00 (11)	2.06 (11)	1.87 (9)	0.77
miR-19a-3p	1.54 (7)	1.90 (11)	0.90 (10)	1.07 (7)	0.40
miR-31-5p	2.91 (7)	18.04 (11)	23.64 (11)	73.11 (10)	0.11
miR-32-5p	0.39 (7)	0.60 (7)	0.18 (7)	0.11 (4)	0.13
miR-99a-5p	2.31 (6)	5.48 (6)	14.35 (5)	3.42 (6)	0.19
miR-200b-3p	0.86 (6)	5.04 (9)	4.70 (6)	18.63 (6)	0.20
miR-215-5p	8.06 (6)	16.01 (6)	3.49 (5)	15.71 (5)	0.32
miR-331-3p	4.67 (7)	12.64 (10)	13.96 (9)	6.50 (4)	0.34
miR-374b-5p	0.88 (7)	0.84 (11)	0.46 (11)	0.51 (10)	0.37
miR-660-5p	4.18 (7)	5.87(11)	3.15 (10)	7.16 (6)	0.73

**Table 1.** Phase I and phase II studies of VPA.

Phase I/II studies				
Author	**Malignancy; N**	**Combination**	**Dosage**	**Response rate (RR)**
Garcia-Manero *et al* [53]	AML/ myelodysplastic syndrome (MDS) (54)	5AZA	50 mg/kg/day per os (PO)	RR 22%
Soriano *et al*, [44]	AML/MDS (53)	All-Trans Retinoic AcidIV: intravenous (ATRA) + 5AZA	50 mg/kg/day PO 7 days	RR 47%
Daud *et al* [54]	Melanoma (39)	Karenitecine	75 mg/kg/day IV d1 to d5	47% stable disease
Rocca *et al* [45]	Melanoma (29)	Dacarbazine + interferon	Serum level of 50 to 125 mg/dL	5% CR; 11% PR; 16% stable disease
Munster *et al* [25]	Solid tumours(44)	Epirrubicine/ 5-fluorouracil, epirrubicin, cyclophosphamide (FEC)	120 mg/kg IV	RR 22% (phase I) RR 68% phase II (breast cancer)
Iwahashi *et al* [17]	Pancreas/biliary(12)	S-1	15 mg kg 2xd PO	8% PR; 83% stable disease
Phase II studies				
Author	**Malignancy; N**	**Combination**	**Dosage**	**Response rate**
Kuendgen *et al* [55]	AML/MDS (75)	ATRA	Serum levels 50 to 100 mcg/mL	RR 5% (AML), 16% (MDS)
Pilatrino *et al* [56]	AML/MDS (20)	ATRA	Serum levels 45 to 100 mcg/mL	Clinical benefit 30%
Candelaria *et al* [57]	Solid tumours (17)	Hidralazine + chemotherapy	40 mg/kg/day	0% CR, 27% PR, 53% stable disease
Raffoux *et al* [58]	AML/MDS (65)	5-AZA + ATRA	35–50 mg/kg for 7 days	RR 26%
Mohammed *et al* [22]	Neuroendocrine tumours (8)	-	500 mg 2xd PO; non significant (NS) 50–100 mcg/mL	12% PR, 50% stable disease
Krauze *et al* [16]	Glioblastoma multiforme (37)	Temozolomide + radiotherapy	25 mg/kg/day	81% stable disease
Issa *et al* [15]	AML/MDS (149)	± Decitabine	50 mg/kg 7 days	RR 51% versus 58% combination

**Table 2.** Patients’ characteristics.

	*N* = 10 (%)
Median age	55 (41–65)
Male	10 (100)
Race	
White	8 (80)
Black	2 (20)
Smoking history	
Current	4 (40)
Former	6 (60)
Pack-years (median)	51 (15–80)
Former alcoholic	10 (100)
Weight loss	
0%–10%	5 (50)
10%–20%	4 (40)
>20%	1 (10)
ECOG	
0	4 (40)
1	6 (60)
OP	10 (100)
Stage	
III	1 (10)
IVA	8 (80)
IVB	1 (10)
p16 immunohistochemistry	
Negative	10 (100)
High-risk HPV	3 (30)
Cisplatin dose	
Cycles (median)	2.5 (1–3)
Median dose (mg/m^2^)	230 (100-300)
Radiotherapy	
70 Gy	10 (100)
Treatment weeks (median)	7.4 (6.9–8.6)

**Table 3.** Treatment related toxicities.

Adverse events	*N* (%)
Grade 3 and 4	8 (89)
Serious adverse events	4 (44)

Event	Grade 1	Grade 2	Grade 3	Grade 4	Total
Anaemia	4 (44)	4 (44)	1 (11)	0	9 (100)
Dysgeusia	2 (22)	2 (22)	0	0	4 (44)
Creatinine elevation	3 (33)	2 (22)	1 (11)	0	6 (67)
Weight loss	3 (33)	4 (44)	0	0	7 (78)
Infection	1 (11)	2 (22)	3 (33)	0	6 (67)
Lymphopenia	2 (22)	0	4 (44)	2 (22)	8 (89)
Oral moniliasis	0	3 (33)	2 (22)	0	5 (56)
Mucositis	1 (11)	6 (67)	0	0	7 (78)
Nausea	3 (33)	1 (11)	2 (22)	0	6 (67)
Neutropenia	2 (22)	0	2 (22)	0	4 (44)
Radiodermitis	5 (56)	1 (11)	0	1 (12)	7 (78)
Somnolence	3 (33)	2 (22)	1 (11)	0	6 (67)
Vomiting	4 (44)	1 (11)	0	0	5 (55)
Xerostomia	5 (56)	0	0	0	5 (56)

**Table 4.** Patients’ outcomes.

	*N* (%)
RR	8 (89)
Complete response	5 (56)
Parcial response	3 (33)
Progressive disease	1 (12)
Non-evaluable	1
Treatment failure	5 (50)
Recurrence/local progression	4 (40)
Systemic progression	2 (20)
Death	5 (50)
Disease	4 (40)
Others	1 (10)
Current living and without disease	5 (50)
Median follow-up (months)	52 (11.2–71.9)
Median OS	44 months

**Table 5.** Main validated deregulated miRs targets related to carcinogenesis in squamous cell carcinoma patients.

miR	Carcinogenesis related targets
miR-31	TP53, Forkhead Box P3 (FOXP3), Ret Proto-Oncogene (RET), ADP Ribosylation Factor 1 (ARF1), MET
miR-let-7b	Myc regulation
miR-222	TP53
miR-145	CCND2, Neurotrophic Receptor Tyrosine Kinase 2 (NTRK2), HDAC2, EGFR, TGFB, VEGFA, CDK6

**Table 6.** miR validation in saliva. Median expression of selected miRs by qPCR.

	HV (N)	Baseline(N)​	*p*
miR-1-3p​	4.57 (8)	3.29 (11)	0.90
miR-let-7a-5p​	1.05 (8)	0.69 (11)	**0.05**
miR-let-7e-5p​	5.28 (5)	1.64 (11)	**0.04**
miR-32-5p​	0.04 (3)	0.67 (7)	0.32
miR-660-5p​	0.66 (8)	0.91(11)	0.90
miR-26b-5p​	1.09 (7)	0.54(11)	0.10
miR-425-5p​	0.10 (8)	0.40(11)	**0.03**
